# Plasma cross-gestational sphingolipidomic analyses reveal potential first trimester biomarkers of preeclampsia

**DOI:** 10.1371/journal.pone.0175118

**Published:** 2017-04-06

**Authors:** Aneta Dobierzewska, Sony Soman, Sebastian E. Illanes, Andrew J. Morris

**Affiliations:** 1 Department of Obstetrics & Gynecology and Laboratory of Reproductive Biology, Faculty of Medicine, Universidad de los Andes, Santiago, Chile; 2 Division of Cardiovascular Medicine and the Gill Heart Institute, University of Kentucky College of Medicine, Lexington, KY, United States of America; 3 Department of Veterans Affairs, Medical Center Lexington, Kentucky, United States of America; Medical University of South Carolina, UNITED STATES

## Abstract

**Introduction:**

Preeclampsia (PE) is a gestational disorder, manifested in the second half of pregnancy by maternal hypertension, proteinuria and generalized edema. PE is a major cause of maternal and fetal morbidity and mortality, accounting for nearly 40% of all premature births worldwide. Bioactive sphingolipids are emerging as key molecules involved in etiopathogenesis of PE, characterized by maternal angiogenic imbalance and symptoms of metabolic syndrome. The aim of this study was to compare the cross-gestational profile of circulating bioactive sphingolipids in maternal plasma from preeclamptic (PE) versus normotensive control (CTL) subjects with the goal of identifying sphingolipids as candidate first trimester biomarkers of PE for early prediction of the disease.

**Methods:**

A prospective cohort of patients was sampled at the first, second and third trimester of pregnancy for each patient (11–14, 22–24, and 32–36 weeks´ gestation). A retrospective stratified study design was used to quantify different classes of sphingolipids in maternal plasma. We used a reverse-phase high-performance liquid chromatography-tandem mass spectrometry (HPLC-ESI-MS/MS) approach for determining different sphingolipid molecular species (sphingosine-1-phosphate (S1P), dihydro-sphingosine-1-phosphate (DH-S1P), sphingomyelins (SM) and ceramides (Cer)) in cross-gestational samples of human plasma from PE (n = 7, 21 plasma samples across pregnancy) and CTL (n = 7, 21 plasma samples across pregnancy) patients.

**Results:**

Plasma levels of angiogenic S1P did not change significantly in control and in preeclamptic patients´ group across gestation. DH-S1P was significantly decreased in second trimester plasma of PE patients in comparison to their first trimester, which could contribute to reduced endothelial barrier observed in PE. The major ceramide species (Cer 16:0 and Cer 24:0) tended to be up-regulated in plasma of control and PE subjects across gestation. The levels of a less abundant plasma ceramide species (Cer 14:0) were significantly lower in first trimester plasma of PE patients when compared with their gestational-matched control samples (p = 0.009). Major plasma sphingomyelin species (SM 16:0, SM 18:1 and SM 24:0) tended to be higher in control pregnancies across gestation. However, in PE patients, SM 16:0, SM 18:0 and SM 18:1 showed significant up-regulation across gestation, pointing to atherogenic properties of the sphingomyelins and particularly the potential contribution of SM 18:0 to the disease development. In addition, two major sphingomyelins, SM 16:0 and SM 18:0, were significantly lower in first trimester plasma of PE patients versus first trimester samples of respective controls (p = 0.007 and p = 0.002, respectively).

**Conclusions:**

Cross-gestational analysis of maternal plasma of preeclamptic and normotensive women identifies differences in the biochemical profile of major sphingolipids (DH-S1P, sphingomyelins and ceramides) between these two groups. In addition, first trimester maternal plasma sphingolipids (Cer 14:0, SM 16:0 and SM 18:0) may serve in the future as early biomarkers of PE occurrence and development.

## Introduction

Preeclampsia (PE), a serious pregnancy-associated disorder, is characterized by high maternal and fetal morbidity and mortality and is a cause of nearly 40% of premature births worldwide [1, 2]. Clinical manifestations of PE include new onset hypertension, proteinuria and edema appearing in the second half of pregnancy in the normotensive woman, and in severe cases it may lead to maternal end-organ dysfunction (HELLP syndrome) [2, 3]. PE very often has future consequences for mothers and children born from preeclamptic pregnancies that includes increased cardiovascular complications or metabolic syndrome and related disorders [4–8]. Despite ongoing research into the characterization of molecular mechanisms that trigger PE, its exact pathogenesis remains incompletely understood. To date there is no clinically available early (first trimester) predictive diagnostics test, which would identify women at risk of developing PE before the onset of the disease in the second half of pregnancy [9, 10]. So far, the only feasible management of preeclamptic pregnancies, is the antenatal control of all pregnant women and earlier interruption of pregnancy when severe PE is diagnosed [9].

To the present time several different biochemical markers to early predict PE have been proposed; markers of endothelial damage (anti-angiogenic markers), like: soluble fms-like tyrosine kinase-1 (sFlt-1), soluble endoglin (sEDG); markers of apoptosis and inflammation; placental protein 13 (PP13), C-reactive protein; markers of placental hypoxia and distress: leptin, hypoxia-inducible factor-1α (HIF-1α), inhibin-A and activin-A, or circulating placental microvesicles (e.g. exosomes) as potential signature nanoparticles [10]. However, none of these biomarkers are sufficiently sensitive and specific to predict PE in the first trimester [10], highlighting the need of more research to find biomarkers related to the pathological processes occurring in early pregnancy in patients that will develop PE.

Recently sphingolipids and sphingolipids-related proteins have been implicated as potential key factors involved in pathogenesis of PE [11, 12] that may serve as early biological sensors of PE development. Two major sphingolipids, ceramide (Cer) and sphingosine-1-phosphate (S1P) and their synthetic/metabolic pathways have been shown to be involved in physiological process of trophoblast differentiation and invasion *in vitro* and uterine angiogenesis, which are severely impaired in PE [13–15]. The imbalance of the so called sphingolipid ¨rheostat¨ has been demonstrated recently in preeclampsia, as the reduced levels of circulating angiogenic S1P and elevated levels of pro-apoptotic ceramides (Cer16:0, Cer18:0, Cer20:0, Cer24:0) were found in third trimester serum of PE patients as compared to normotensive controls [11]. Further, the lipidomic analysis of human term umbilical cord veins (UCV) from PE and control patients revealed aberrant sphingolipids composition as preeclampsia was found to be associated with significant decrease in total sphingomyelins (SM) and total ceramides (Cer) and increase in sphinganine and S1P [16].

Mass spectrometry-based lipidomics of sphingolipids (sphingolipidomics) is emerging as a very accurate and sensitive platform for discovery of disease biomarkers [17, 18]. Circulating sphingolipids are present in human blood and therefore the liquid biopsy is non-invasive and easy source to isolate, extract and measure these molecules [19]. Angiogenic S1P is a blood borne lipid mediator, and is associated with lipoproteins such as HDL and with albumin [20, 21]. The major source of plasma S1P are red blood cells, vascular endothelial cells (ECs), and activated platelets [20, 22–23]. Sphingomyelins (SM) and ceramides (Cer) are mainly associated with low-density lipoproteins (LDL), pointing to atherogenic properties of these sphingolipids [24].

This study was aimed to characterize the metabolic profile of three major sphingolipids (sphingosine-1-phosphate, sphingomyelins and ceramides) in plasma of preeclamptic and control normotensive patients during early, mid and late gestation and to determine if preeclampsia is associated with aberrant sphingolipid profile when compared to controls and if there are any specific sphingolipid molecular species which could be potential candidates as first trimester signature molecules of PE onset and development.

## Materials and methods

### Study design

This study was approved by Ethics Committees of Universidad de los Andes and Hospital Parroquial de San Bernando (Santiago Chile), and written informed consent was obtained from all study subjects for collection of blood/plasma samples. Women (n = 500) were recruited between January 2008 and December 2010 with informed, written consent by research midwives from the Hospital Parroquial de San Bernardo, Santiago, Chile. Serial blood samples from fasted patients (BD Vacutainer PLUS Tubes, EDTA) were collected at 11–14 (early), 22–24 (mid), and 32–36 (late) weeks of gestation.

Clinical variables, and pregnancy outcomes were recorded. A retrospectively stratified study was designed involving normal healthy pregnant women (n = 7) and patients with preeclampsia (PE) (n = 7). Both groups consisted of women with singleton gestation and none of them took multivitamins and aspirin during pregnancy. Preeclampsia was diagnosed based on the presence of hypertension (arterial pressure (AP) higher or equal to AP 140/90 mmHg on two occasions separated by 6h or higher or equal to 160/110 mmHg in one occasion) that occurred after 20 weeks of gestation, and proteinuria (300mg/24h). Controls, who did not differ in racial origin from PE patients, were healthy subjects without pregnancy complications or chronic medical problems.

### Isolation of plasma from maternal circulation

Maternal blood from overnight fasted patients was collected in BD Vacutainer glass plasma tubes (6ml) with EDTA as anticoagulant. Blood was collected at three different points of gestation for each patient (1^st^, 2^nd^ and 3^rd^ trimester). With plasma collection, the need to properly fill-up the tubes during collection and adequate mixing of the sample into the additive were important as to provide the optimum blood/anticoagulant ratio. After collection, blood samples were kept at room temperature for no more than 12 hours and then were centrifuged at 1500xg for 15min. After plasma fractions were separated, they were aliquoted in 0.5 or 1ml aliquots and stored in -80C until lipid extraction and analysis.

### Lipid extraction and sphingolipid analysis using tandem mass spectrometry

Total lipid extracts from 50 *μ*l of maternal plasma were obtained using acidified organic solvents as described previously [25] with the addition of S1P (sphingosine-1-phosphate), Cer (ceramide) and SM (sphingomyelin) internal standards containing a C17:0 fatty acid (Avanti Polar Lipids, Albaster, AL, USA). The lipid containing lower organic phase was evaporated to dryness under N2 gas using a Zymark Turbovap and reconstituted in methanol (0.1ml). Molecular species of S1P, SM and Cer were detected by monitoring species-specific precursor product ion pairs by HPLC–ESI (electrospray ionization) tandem MS using 4000 Q-Trap hybrid linear ion trap triple-quadrupole mass spectrometer as described previously [26] with minor modifications. The methods employed an AB Sciex-4000 triple quadrupole mass spectrometer (AB Sciex, Framingham, MA, http://www.sciex.com) operated in multiple reaction monitoring (MRM) mode coupled with a Shimadzu UFLC (Shimadzu Corp., Kyoto, Japan, http://www.shimadzu.com) system. The mass spectrometer was operated in positive electrospray ionization mode with optimized declustering potentials, collision energies and exit potentials. The flow rate was 0.5 ml/minute with a column temperature of 30°C. The sample injection volume was 10 μl. Ceramides and sphingomyelins were separated using a Waters X Terra C8 column (3.5 μm, 3.0 x 100 mm). The mobile phase consisted of 61/39/0.5 Methanol/5mM Ammonium formate/Formic acid as solvent A and 90/10/0.5/0.5 Acetonitrile/ Chloroform/5mM Ammonium formate/Formic acid as solvent B. Analysis was done by maintaining 0% B for 3 minutes, increasing to 100% B in the next 5 minutes, maintaining at 100% B for 2 minutes, followed by column equilibration to initial conditions in the next 3 minutes. For analysis of S1P and DH-S1P, separation was done using an Agilent Zorbax Eclipse XDB C8 column (4.6 × 150 mm, 5 μM). The mobile phase consisted of 75/25 of Methanol/Water with Formic acid (0.5%) and 5mM Ammonium formate (0.1%) as solvent A and 99/1 of Methanol/Water with Formic acid (0.5%) and 5mM Ammonium formate (0.1%) as solvent B. Separation was achieved using a gradient of 0% B for 1 minute increasing to 100% B over the next 1 minute and maintaining at 100% B for the next 10 minutes. This was followed by equilibrating the column to the initial conditions in 3 minutes. S1P and DH-S1P were quantified by comparison to calibration curves produced using S1P standards (Avanti Polar Lipids) (independently measured by phosphorus analysis after perchloric acid digestion)), after correcting for recovery with reference to the internal standard. Likewise, sphingomyelins containing palmitic (C16:0), stearic (C18:0), oleic (C18:1) and lignoceric (C24:0) acids and ceramides with myristic (C14:0), palmitic (C16:0), stearic (C18:0), arachidic (C20:0), behenic (C22:0), lignoceric (C24:0) and nervonic (C24:1) acids were measured.

The lipidomic measurements were performed at Small Molecule Mass Spectrometry Core Laboratory at University of Kentucky.

### Statistical analysis

All data are represented as mean (±) SD from plasma cross-gestational samples of 7 normotensive (CTL) controls (total 21 plasma samples) and 7 preeclamptic (PE) patients (total 21 plasma samples). Statistical significance between the groups (normotensive versus preeclamptic patients, e.g. 1^st^ trimester of CTL group versus 1^st^ trimester of PE group) was determined by nonparametric Mann-Whitney U-test. For statistical analyses inside the control (CTL) or PE group (e.g. 1^st^ vs 2^nd^ trimester of CTL group or 1^st^ vs 2^nd^ trimester of PE group, etc) was used paired Wilcoxon test. All graphs and statistical analyses were performed using GraphPad Prism software (San Diego, CA). Data were considered statistically significant at two-tailed *p value ≤ 0.05 and **p ≤ 0.01.

## Results

### Maternal characteristics

A total of fourteen women with either no pregnancy complications (n = 7, 21 plasma samples across pregnancy) or those who developed preeclampsia (n = 7, 21 plasma samples across pregnancy) were included in the study. The clinical characteristics of preeclamptic (PE) and control normotensive (CTL) subjects are presented in Table 1. Women who developed PE showed a significantly higher systolic and diastolic arterial pressure (p = 0.0006) as compared to control subjects. There were no significant differences in maternal and gestational age at delivery between control and preeclamptic patients. Additionally there was no significant difference in fetus weight between normotensive and PE group, however in few cases of preeclamptic pregnancies the newborns weight was below 3000g when compared with control pregnancies. Although there was no statistical difference in body mass index (BMI) between both groups; despite of few individuals with normal BMI in each group, the majority of the patients qualified as overweight (BMI: 25–30) or obese (BMI more than 30).

**Table 1:** Clinical characteristics of patient and delivery.

Variable	Normotensive control group CTL (n = 7)	Preeclamptic group PE (n = 7)	*P* value
Maternal age (yr)	23.3 ± 3.5	28.7 ± 7.2	*p* = 0.133
Gestational age of delivery (wk)	38.2 ± 1.9	37.9 ± 1.2	*p* = 0.828
Maternal body mass index BMI	29.2 ± 6.2	31.2 ± 5.8	*p* = 0.71
Systolic pressure (mmHg)	112.9 ± 11.1	145.7 ± 7.3	*p* = 0.0006**
Diastolic pressure (mmHg)	68.6 ± 6.9	96.4 ± 7.5	*p* = 0.0006**
Newborn weight (g)	3592 ± 235.9	3285 ± 558.2	*p* = 0.383
Fetal sex (male/female)	(6/1)	(5/2)	
Mode of delivery (CS/VD)	(3/4)	(4/3)	

Values are given as a mean ±SD. Statistical significance was assessed by Mann-Whitney test.

** *p*≤ 0.01

### S1P and DH-S1P profile in maternal plasma of preeclamptic (PE) and control normotensive (CTL) patients

The blood samples of each fasted individual were obtained at three points of gestation, early (1^st^ trimester), mid (2^nd^ trimester) and late (3^rd^ trimester). Lipids from maternal plasma were isolated as described in Materials & Methods. Reverse-phase HPLC-ESI-tandem mass spectrometry analysis was used to determine the levels of different sphingolipids in plasma cross-gestational samples of PE patients versus normotensive controls. Analyzed sphingolipids included: sphingosine-1-phosphate (S1P), dihydro-sphingosine-1-phosphate (DH-S1P), molecular species of ceramide and sphingomyelin with specific fatty-acid content. The major fatty acids found in plasma sphingolipids were palmitic (C16:0), stearic (C18:0), oleic (C18:1), lignoceric (C24:0) and nervonic (C24:1) acid.

**Fig 1:**
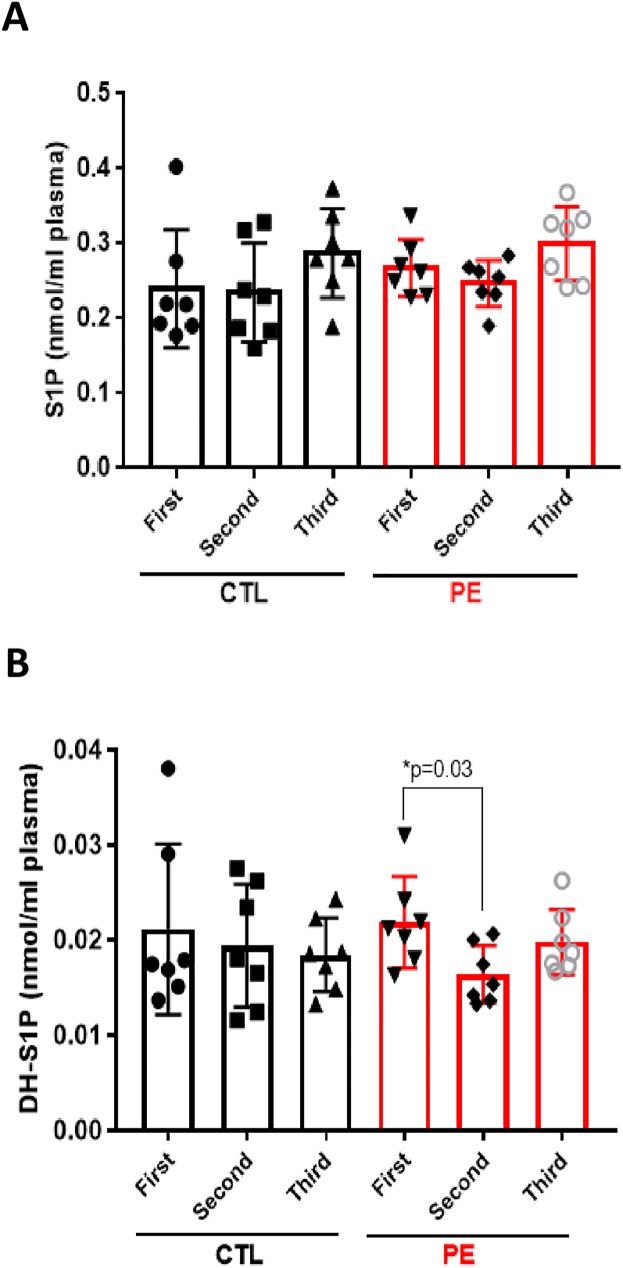
S1P and DH-S1P profile in maternal plasma of PE and CTL patients. Each target analyte: sphingosine-1-phosphate (S1P) **(A)** and dihydro-sphingosine-1-phosphate (DH-S1P) **(B)** were analyzed by reversed-phase HPLC-MS/MS and identified by its specific parent-daughter ion mass transition and retention time. Plasma S1P and DH-S1P levels were measured in 7 normotensive control (CTL) and 7 preeclamptic (PE) patients across gestation: first-, second- and third trimester. Data expressed as means ± SD. Statistical differences detected between groups are indicated with an asterisk (n = 7 for each group, p<0.05).

Plasma levels of angiogenic S1P did not changed significantly through gestation in both groups (PE: 0.267–0.3 nmol/ml plasma vs CTL: 0.239–0.287 nmol/ml plasma) (Fig 1A). Our results differ from previous observations of *Melland-Smith et al* [11], as they detected significant reduction of late-gestation (3^rd^ trimester) serum S1P in PE versus control subjects. We measured also the levels of dihydro-S1P (DH-S1P) (Fig 1B), which has been recently linked to enhancement of endothelial barrier [27]. DH-S1P did not changed in control group across gestation (0.021–0.018 nmol/ml plasma). In preeclamptic group there was significant decrease (p = 0.03) of DH-S1P in the 2^nd^ trimester (0.016 nmol/ml plasma) when compared to their 1^st^ trimester (0.021 nmol/ml plasma), pointing eventually to endothelial damage (reduced endothelial barrier) occurring in PE (Fig 1B).

Plasma levels of S1P and DH-S1P measured in our clinical samples were comparable with plasma levels of these two sphingolipids reported previously in a study of basic metabolic profile in a group of males and non-pregnant females (0.31 uM (S1P) and 0.04 uM (DH-S1P), respectively) [19].

### Sphingomyelin profile in maternal plasma of PE and CTL patients

**Fig 2:**
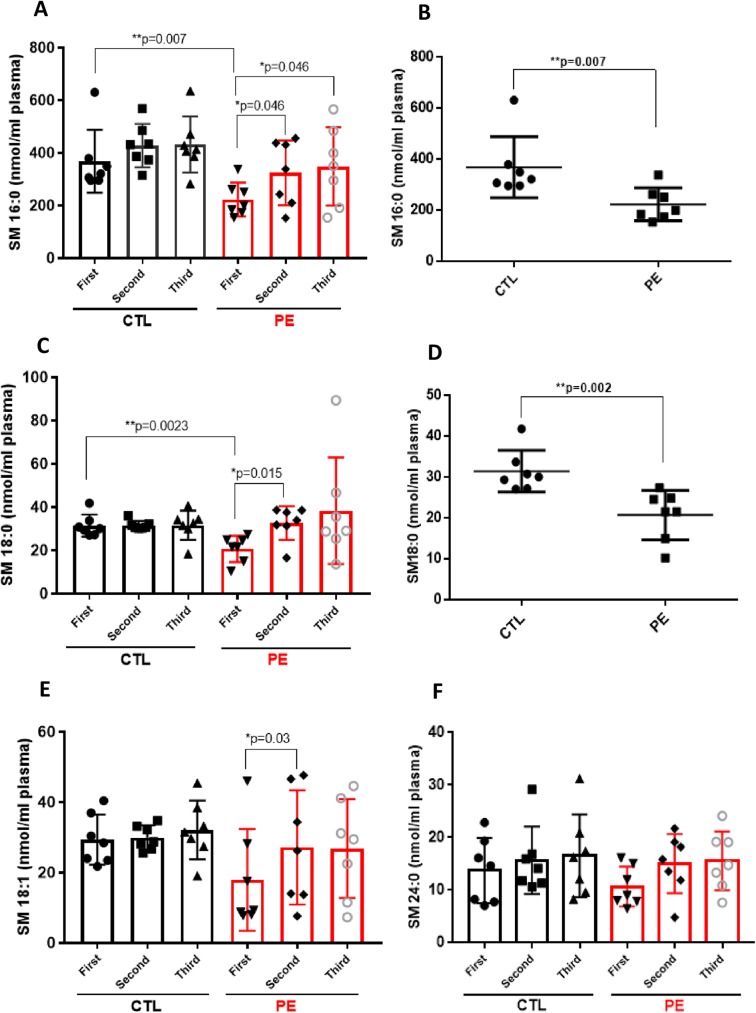
Sphingomyelin profile in maternal plasma of PE and CTL patients. Each target analyte: (sphingomyelin (SM) species) was analyzed by reversed-phase HPLC-MS/MS and identified by its specific parent-daughter ion mass transition and retention time. Different sphingomyelin species: SM 16:0 **(A)**, SM 16:0 in 1^st^ trimester plasma of PE and CTL patients **(B)**, SM 18:0 **(C)**, SM 18:0 in 1^st^ trimester plasma of PE and CTL patients **(D)**, SM 18:1 **(E)** and SM 24:0 **(F)** were measured in plasma of 7 normotensive control (CTL) and 7 preeclamptic (PE) patients across gestation: first-, second- and third trimester. Data expressed as means ± SD. Statistical differences detected between groups are indicated with an asterisk (n = 7 for each group, p<0.05).

We next determined the concentrations of various sphingomyelin species in the same samples (Fig 2). In line with the previous observations [19], sphingomyelin was the dominant circulating sphingolipid. The most prominent sphingomyelin was SM 16:0, showing up-regulation in both groups across gestation, but with statistical significance only in PE group (1^st^ vs 2^nd^ trimester of PE and 1^st^ vs 3^rd^ trimester of PE, p = 0.046). The concentration of SM 16:0 in PE group ranged from 222.7 ± 64.17 up to 348.7 ± 149.1 nmol/ml plasma and in controls (CTL) was from 368 ± 119.7–432.3 ± 106.6 nmol/ml plasma (Fig 2A). When comparing PE and control patients, SM 16:0 was significantly reduced (p = 0.007) in 1^st^ trimester plasma of PE subjects versus controls (222.7 ± 64.17 vs. 368 ± 119.7 nmol/ml) (Fig 2B). The other SM species analyzed included: SM 18:0, SM 18:1 and SM 24:0, which constituted the next most abundant sphingomyelin subpopulations (Fig 2C–2F). First trimester plasma levels of SM 18:0 were also significantly decreased (p = 0.002) in PE group (20.7 ± 6.04 nmol/ml) when compared with controls (31.41 ± 6.04 nmol/ml) (Fig 2D). However, SM 18:0 in PE patients was significantly elevated through gestation (from 20.7 ± 6.04 up to 38.32 ± 14.64 nmol/ml plasma, p = 0.015) (Fig 2C), pointing to atherogenic properties of this particular sphingomyelin [24]. In control group, SM 18:0 remained unchanged in early, mid and late gestation (ca. 31.6 nmol/ml plasma). In regards to SM 18:1 there were no statistical differences between PE and CTL group, however there was significant up-regulation of SM 18:1 in PE group between the 1^st^ and 2^nd^ trimester of pregnancy (p = 0.03). Levels of SM 24:0 did not change significantly in any of the group or between the groups across gestation (Fig 2E and 2F).

Levels of plasma sphingomyelins measured in our clinical samples were comparable to those as previously published [19]; however, the levels of SM 16:0 in plasma of our fasted PE and CTL patients (222.7–427.5 nmol/ml) were higher as of those previously reported in fasted subjects (males and non-pregnant females - 100uM). This difference could have its origin as influence of the pregnancy on basic SM 16:0 profile or could stem from differences in patients´ ethnicity.

### Ceramide profile in maternal plasma of PE and CTL patients

**Fig 3:**
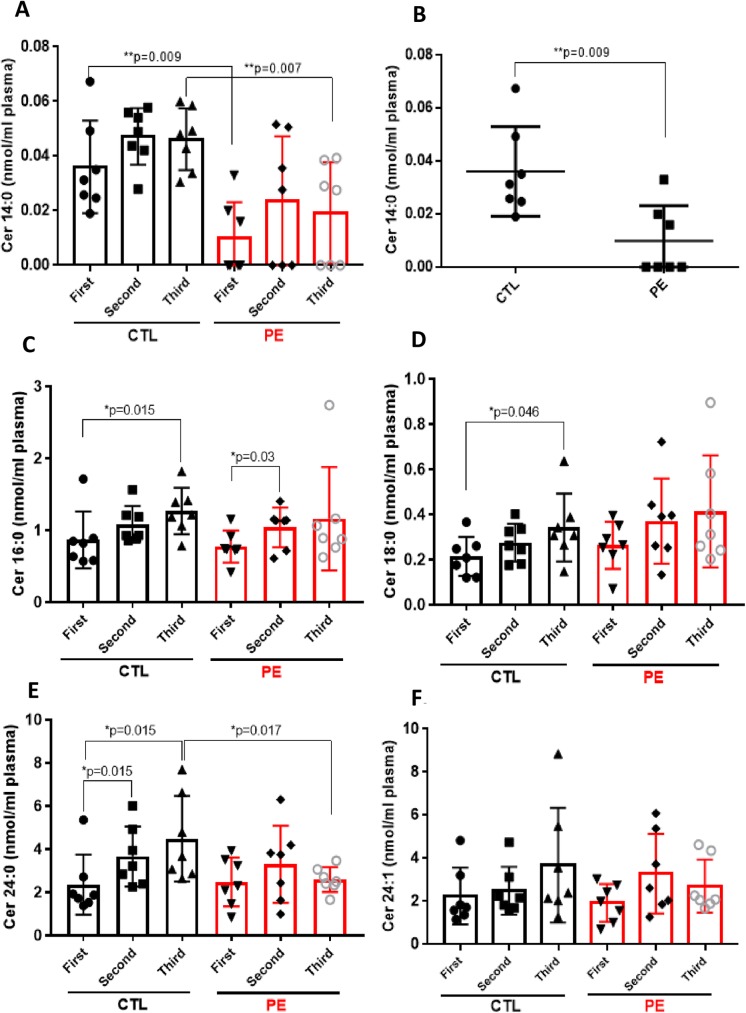
Ceramide profile in maternal plasma of PE and CTL patients. Each target analyte: (ceramide (Cer) species) was analyzed by reversed-phase HPLC-MS/MS and identified by its specific parent-daughter ion mass transition and retention time. Different ceramide species: Cer 14:0 **(A)**, Cer 14:0 in 1^st^ trimester plasma of PE and CTL patients **(B)**, Cer 16:0 **(C)**, Cer 18:0 **(D)**, Cer 24:0 **(E)** and SM 24:1 **(F)** were measured in plasma of 7 normotensive control (CTL) and 7 preeclamptic (PE) patients across gestation: first-, second- and third trimester. Data expressed as means ± SD. Statistical differences detected between groups are indicated with an asterisk (n = 7 for each group, p<0.05).

We also analyzed the molecular species of ceramide (Cer) in PE and CTL plasma samples. As previously reported [19], the most dominant ceramide species were Cer 24:0 and Cer 24:1, followed by Cer 16:0 and Cer 18:0 (Fig 3). The less abundant Cer 14:0 was significantly decreased in 1^st^ and 3^rd^ trimester plasma of PE patients when compared with control subjects during the same gestational periods (1^st^ trimester–PE: 0.009 ± 0.013 nmol/ml vs CTL: 0.036 ± 0.016 nmol/ml, p = 0.009 and 3^rd^ trimester–PE: 0.019 ± 0.018 nmol/ml vs CTL: 0.046 ± 0.011 nmol/ml, p = 0.007, respectively) (Fig 3A and 3B). The second most abundant Cer 16:0 was increased significantly in control plasma samples across gestation (Fig 3C). Plasma levels of Cer 16:0 in controls ranged from 0.864 ± 0.395 nmol/ml up to 1.266 ± 0.322 nmol/ml (1^st^ vs 3^rd^ trimester of CTL, p = 0.015) (Fig 3C). Cer 16:0 also increased in cross-gestational PE plasma samples, with statistical significance between the 1^st^ and 2^nd^ trimester of pregnancy (p = 0.03) (Fig 3C). Mean plasma levels of Cer 18:0 showed tendency to be up-regulated in both groups across gestation, with significant differences between the 1^st^ and 3^rd^ trimester in control group (CTL: from 0.213 ± 0.086 up to 0.342 ± 0.15 nmol/ml and PE: from 0.263 ± 0.104 up to 0.413 ± 0.248 nmol/ml) (Fig 3D). The most abundant Cer 24:0 was increased significantly in control plasma samples across gestation, from 2.353 ± 1.39 up to 4.477 ± 1.98 nmol/ml (p = 0.015) (Fig 3E). Cer 24:0 in PE group did not changed significantly through gestation, however there was significant down-regulation of Cer 24:0 in 3^rd^ trimester PE plasma samples (2.588 ± 0.574 nmol/ml) when compared to their matched-gestational controls (4.477 ± 1.986 nmol/ml, p = 0.017) (Fig 3E).

As reduced levels of circulating Cer 24:0 were previously linked to liver cirrhosis [28], the observed by us reduced levels of Cer 24:0 in PE patients could point to signs of liver damage in preeclampsia. Finally the levels of other abundant circulating ceramide, Cer 24:1 did not changed significantly in both PE and CTL plasma samples throughout gestation (Fig 3F).

## Discussion

Recent studies demonstrate the feasibility of mass spectrometry-based lipidomics as a tool to identify and quantitate circulating bioactive lipids in biofluids of healthy and diseased patients [29]. Because bioactive lipids are emerging as signature molecules of development and progression of many diseases [17, 18, 29], in the near future lipidomics could become a standard clinical diagnostic tool in molecular medicine.

This study was conducted to determine the basic sphingolipid profile in plasma samples of control normotensive and preeclamptic patients across gestation with the goal of identifying first trimester sphingolipids as candidates as potential biomarkers for early prediction of PE.

In our cross-gestational study, we found that circulating angiogenic S1P did not change significantly across gestation in neither the control nor the preeclamptic group. Dihydro-S1P followed the similar pattern, only with the exception that it was decreased in plasma of PE patients in the 2^nd^ trimester when compared with the 1^st^ trimester. In contrast, major ceramide species (Cer 16:0, Cer 18:0 and Cer 24:0) were significantly elevated through gestation in plasma of control patients, while in PE pregnancies only Cer 16:0 was significantly up-regulated between the 1^st^ and 2^nd^ trimester of pregnancy. The levels of less abundant ceramide (Cer 14:0) were significantly lower in 1^st^ trimester plasma of PE patients when compared with 1^st^ trimester control samples (p = 0.009). Plasma concentrations of major sphingomyelin species (SM 16:0, SM 18:0, SM 18:1 and SM 24:0) tended to be higher in the control group across gestation than in the case group. However, in PE patients, sphingomyelins–SM 16:0, SM 18:0, and SM 18:1 were significantly up-regulated across gestation. Additionally, levels of two major sphingomyelins, SM 16:0 and SM 18:0, were significantly lower in 1^st^ trimester plasma of PE patients as compared to 1^st^ trimester samples of respective controls (p = 0.007 and p = 0.002, respectively).

Recent published reports about sphingolipids and pregnancy, confirm the important role of sphingosine kinase-1 (SPHK1)/sphingosine-1-phosphate (S1P) pathway in the reproductive system and physiopathology of preeclampsia [11, 12]. During mouse pregnancy, the levels of angiogenic SPHK1, an enzyme synthesizing S1P from the precursor sphingolipid—sphingosine [30, 31], and as well angiogenic S1P receptors (S1PR1, -2, -3) are significantly up-regulated during early gestation, a period of time in which extensive angiogenesis is observed in the uterus [32]. Reagarding preeclampsia, recent results from our laboratory have shown the significant decrease of placental angiogenic SPHK1 and S1P receptors (S1PR1 and -3) in term placentae of PE patients when compared to controls [12]. In contrast, the lipidomic data presented in this paper do not provide evidence for regulation of plasma S1P levels across gestation in normal and as well as PE pregnancies. The exception to these observations was a tendency of increased S1P levels in 3^rd^ trimester plasma in both groups as compared to their earlier gestational periods (CTL: from 0.239 (1^st^) up to 0.287 nmol/ml (3^rd^) and PE: from 0.267 (1^st^) up to 0.3 nmol/ml (3^rd^), respectively). As in mouse pregnancy SPHK/S1P pathway has been shown to be crucial in early uterine decidualization and uterine angiogenesis [15], in normal human pregnancies one would expect to observe increased levels of circulating S1P during the early and mid-gestation as a marker of early endometrial/placental vascularization. Moreover, in contrast to lipidomic results published by *Melland-Smith et al* describing reduced S1P levels in 3^rd^ trimester serum of PE patients versus their controls [11], we observed lack of decrease of plasma S1P in PE patients at any of the gestational points. The decrease of circulating angiogenic S1P in PE patients compared to their matched controls, as reported by these authors [11] might point to impaired angiogenesis in PE. The reasons for the observed discrepancy between our and *Melland-Smith et al*. studies could be the larger number of subjects used by the other group [11], or as reported before, the difference in S1P levels between serum and plasma [19]. Moreover, it is also important to take into consideration the differences in patients’ clinical characteristics. In the study of *Melland-Smith et al*, a strong difference regarding average newborns’ weights between preeclamptic and normotensive pregnancies could be noted (PE: 1249 ± 423 g versus CTL: 3712 ± 231g). This would suggest that these preeclamptic placentae were strongly ischemic and hypoxic as the consequence of impaired angiogenesis occurring early in gestation, thus potentially resulting in observed reduced maternal circulating levels of S1P in PE subjects [11]. Regarding our patients, there was no significant difference in fetus weight between preeclamptic and control group (see Table 1).

In addition to S1P, our data revealed reduced plasma content of DH-S1P in the 2^nd^ trimester of PE patients as compared to their 1^st^ trimester, which points eventually to enhanced endothelial damage in PE; as DH-S1P similarly to S1P, has been reported to enhance endothelial barrier [27, 33]. Also previously it has been demonstrated that human DH-S1P serum levels inversely correlate with occurrence of ischemic heart disease (IHD); this may link the reduced plasma DH-S1P in PE patients with the cardiovascular complications appearing later with the progress of the disease [27].

The recent lipidomic studies of *Melland-Smith et al*, point to a possible imbalance of the sphingolipid rheostat in PE based on the elevated levels of ceramides (Cer 16:0, Cer 18:0, Cer 20:0, Cer 24:0) and the reduced levels of S1P that were found in term serum of PE patients as compared with their matched controls [11]. In our cross-gestational plasma samples of PE and normotensive patients, we found that major ceramide species (Cer 16:0, Cer 18:0, Cer 24:0) were elevated consistently across gestation in both groups, except significant reduction of Cer 24:0 in 3^rd^ trimester plasma of PE subjects versus 3^rd^ trimester plasma of controls (p = 0.017). The observed increased ceramide levels in normal pregnancy through gestation could be a consequence of trophoblastic apoptotic shedding as a result of normal syncytial fusion of villous trophoblasts increasing with the gestational age [34, 35]. In contrast, in relation to preeclampsia, as reported previously there is more aponecrotic than apoptotic syncytiotrophoblast shedding [36, 37], which might account for the lack of significant up-regulation of ceramides in PE versus control plasma samples observed in our cohort. In fact, several ceramide-synthesizing enzymes (e.g. acid sphingomyelinase and ceramide kinase) have been linked to trophoblast syncytialization process *in vitro* [13]; however, in regards to preeclampsia it was reported previously that trophoblast cultures from PE pregnancies have rather moderate increase in syncytialization when compared with normal pregnancies [38].

As we discussed above, decreased levels of Cer 24:0 found in 3^rd^ trimester plasma of PE patients versus controls may point to liver damage and cardiovascular complications in preeclampsia. Hence, the reduced levels of circulating Cer 24:0 were found before to be associated with liver cirrhosis [28] and ischemic heart disease (IHD) [27]. We found also that concentration of less abundant ceramide (Cer 14:0) was significantly lower (p = 0.009) in 1^st^ trimester plasma of PE patients (0.0098 ± 0.013 nmol/ml) as compared with controls (0.036 ± 0.016 nmol/ml), pointing eventually to the potential role of Cer 14:0 as an early (1^st^ trimester) biomarker of preeclampsia. As ceramide and their synthesizing enzymes (e.g. ceramide synthases) have been shown previously to be significantly up-regulated in the mice uterus during early gestation [15, 39], in the future the role of Cer 14:0 in the normal human pregnancy and etiopathogenesis of preeclampsia needs to be further explored.

Altered levels of tissue sphingomyelins (SM) have been observed in preeclamptic patients, because higher levels of SM were found in umbilical cord arteries of preeclamptic versus normotensive women [40] and as well as in syncytiotrophoblast microvesicles (STMV) derived from the supernatants of term placental villous explants of PE cases [41]. In contrast to the previous reports, in umbilical cord veins of fetuses from PE subjects, reduced levels of sphingomyelins were detected when compared with controls [16]. Our lipidomic analysis show that all plasma sphingomyelin species we have measured (SM 16:0, SM 18:0, SM 18:1 and SM 24:0), had tendency to be up-regulated across gestation in both groups, preeclamptic and control. Although plasma cross-gestational average values for two sphingomyelins (SM 16:0 and SM 18:1) were higher in control than in PE group, both SM 16:0, SM 18:1 and additionally SM 18:0 were significantly up-regulated in PE group across gestation. SM 18:0 reached average 38.32 nmol/ml in 3^rd^ trimester plasma of PE patients as compared to 31.65 nmol/ml in 3^rd^ trimester of control group. We observed increased levels of plasma SM 18:0 during late gestation in preeclamptic patients which is in line with previous reports describing elevated levels of SM 18:0 in term plasma of early-onset preeclamptic patients [42] and as well as in human PE placentae when compared with control subjects [43]. Recent studies have demonstrated that serum elevation of this particular class of sphingomyelin positively correlates with parameters of insulin resistance and liver function in obese adults [44] and correlates with markers of NF-kB activation and thus markers of intracellular inflammation [44]. It is well-documented in the literature that increased high plasma sphingomyelin levels are associated with subclinical atherosclerosis and coronary artery disease [24]. This may point that sphingomyelins, in particular SM 18:0 might serve not only as atherogenic marker of PE development and progression, but also the marker of cardiovascular complications developed later in the lives of the women who had before preeclamptic pregnancies.

In relation to sphingomyelins, our study identifies two potential early markers of preeclampsia (SM 16:0 and SM 18:0) to be down-regulated in 1^st^ trimester plasma of PE patients as compared with controls (p = 0.007 and p = 0.023, respectively).

In conclusion, to our knowledge, this is the first study to describe longitudinal changes in concentrations of the major sphingolipids in maternal plasma of preeclamptic and normotensive women. Moreover, this study reveals three potential sphingolipid candidates (SM 16:0, SM 18:0 and Cer 14:0) which may serve in the future as early biomarkers of PE, which we found to be significantly down-regulated in 1^st^ trimester plasma of PE patients versus controls. To assure the sensitivity and specificity of these potential markers, the larger patient cohort needs to be screened. In the future, early 1^st^ trimester and highly-sensitive markers of PE would allow to earlier identify women at risk of developing PE. This would enable the earlier antenatal control and treatment of the patients (administration of low-dose aspirin, antenatal corticosteroids for fetal lung maturation) [45] and thus would greatly reduce maternal and fetal morbidity or/and mortality associated with this disease.

## Conclusions

Preeclampsia, a major obstetric complication during pregnancy and a leading cause of maternal and fetal morbidity and mortality, is hard to predict clinically because of lack of the early highly-specific and highly-sensitive biochemical markers that would predict PE onset and development.

This study examines differential cross-gestational sphingolipid patterns in maternal plasma from preeclamptic and normal pregnant women and identifies three plasma sphingolipids (ceramide 14:0, sphingomyelin 16:0, sphingomyelin 18:0) as potential first trimester biomarker candidates of this disease. In the future, sphingolipids may prove to become early biomarkers of preeclampsia, and together with mass-spectrometry-based lipidomics may become a part of routine prenatal diagnostic test early in pregnancy. This would allow identify the patients at risk of developing PE thus allowing their timely prenatal management and treatment, and in consequence would reduce the severity of maternal/fetal complications associated with PE.
